# GC-MS profiling and DPPH radical scavenging activity of the bark of Tampoi ( *Baccaurea macrocarpa*)

**DOI:** 10.12688/f1000research.16643.2

**Published:** 2019-12-12

**Authors:** Erwin Erwin, Widar Ristiyani Pusparohmana, Indah Permata Sari, Rita Hairani, Usman Usman

**Affiliations:** 1 Department of Chemistry, Faculty of Mathematics and Natural Sciences Mulawarman University, Samarinda, East Kalimantan, 75123, Indonesia; 2 Study Program of Chemical Education, Faculty of Teacher Trainer and Education, Samarinda, East Kalimantan, 75242, Indonesia

**Keywords:** Tampoi, Baccaurea macrocarpa, toxicity, BSLT, antioxidant, DPPH

## Abstract

***Background*** *:* Tampoi ( *Baccaurea macrocarpa*) is a tropical rainforest plant that produces edible fruit and is native to Southeast Asia, especially East Kalimantan, Indonesia. Previous research showed that Tampoi potentially can be developed as a drug. It was reported that the extract of Tampoi fruit displayed antioxidant activity, which was correlated with its phenolic and flavonoid substances. There is no information about the antioxidant activity of other parts of this plant, such as the bark, which might also have this kind of activity. Therefore, the aim of this study was to evaluate the phytochemical using GC-MS analysis, toxicity againt *Artemia salina*, and antioxidant activity with DPPH radical scavenging method of the bark of Tampoi.

***Methods***: The bark of Tampoi was extracted with methanol and concentrated using rotary evaporator to obtain the methanol extract of the bark. Secondary metabolites of this extract was determined using phytochemical analysis. Afterward, the methanol extract was tested for its toxicity using brine shrimp lethality test and antioxidant activity using the 2,2-diphenyl-1-picrylhydrazyl method.

***Results*** *:* Phytochemical evaluation results showed that the methanol extract of bark of this plant contains several secondary metabolites including alkaloids, flavonoids, phenolics, steroids, and triterpenoids. The toxicity test displayed no toxic property due to a LC _50 _value above 1000 ppm. For antioxidant activity, the result exhibited that the methanol extract of bark of this plant could be categorized as an active extract with IC _50_ value of 11.15 ppm. Moreover, based on gas chromatography-mass spectrometer analysis, there are 37 isolated compounds from the bark, one of which is methylparaben, a phenolic predicted to act as an antioxidant.

***Conclusion*:** The results obtained in this research demonstrated that the bark of Tampoi ( *B. macrocarpa*) has potential as an antioxidant.

## Introduction

Indonesia is a mega-diverse country in terms of biodiversity that is flanked by the Indian and Pacific Oceans. Indonesia's biodiversity encompasses the diversity of living things both on land and sea ^1^. Indonesia, especially East Kalimantan, has very extensive tropical rainforest, which is a habitat for much biodiversity. Various types of plants have long been utilized by the community as traditional medicines. The utilization of natural products as an alternative medicine is increasing because natural ingredients are believed to be safer than synthetic substances, i.e. contain toxic chemicals that only can be found in modern medicines, which are linked to toxicity ^2^.

Among plants, the genus of *Baccaurea* have interesting biological activities and bark, fruits and leaves of several species are used for medicine such as *B. motleyana* (Rambai) for stomachache and sore eyes, *B. brevipes* for the regulation of menstruation, and *B. lanceolata* against stomach-ache ^3, 4^. The *B. angulata* has been reported as a potential functional food with effective antioxidant ^5^, anti-inflammatory, anti-atherogenic, and hypocholesteromia activities ^6^. Other research has also investigated the biological activity of other species of this genus, i.e. *B. lanceolata* and *B. macrocarpa*. It was reported that the fruits of *B. macrocarpa* exhibited the highest antioxidant activity compared with *B. lanceolata*, which significantly correlated with the phenolic and flavonoid contents ^7^.

The *B. macrocarpa* is one of the typical plants of East Kalimantan, Indonesia and the edible fruits is a source of additional nutrients and known as Tampoi. Tampoi fruit skin has high antibacterial inhibitory effects on the growth of *S. aureus* and *E. coli.*, and it was toxic to Artemia salina ^8, 9^. Until now, the information about the antioxidant activity of other parts of this plant such as the bark of Tampoi has not been reported yet. Hence, the present research was conducted to investigate the phytochemical, toxicity, and antioxidant activity of the bark of Tampoi ( *B. macrocarpa*). Furthermore, the gas chromatography-mass spectrometer (GC-MS) analysis was performed to obtain information about the kinds of compounds contained.

## Methods

### Extraction

Extraction was carried out as described previously by Erwin *et al.* (2014) ^10^. The bark of Tampoi ( *B. macrocarpa*) was dried for one week at room temperature and ground to a powder. The powder was extracted using a maceration method by soaking in methanol for 24 hours at room temperature, which was repeated three times. Afterwards, the extract solution was filtered by filter paper and the solvent was evaporated under vacuum using a rotary evaporator (Buchi R II) at 45°C and 1 atm, to obtain the methanol extract of bark of Tampoi.

### Phytochemical evaluation

Phytochemical evaluation was performed to investigate the secondary metabolites contents of the methanol extract of bark of Tampoi ( *B. macrocarpa*), including alkaloids, flavonoids, phenolics, steroids, triterpenoids, and saponins, as described previously ^11^. The presence of secondary metabolites was identified by observing the changing color of the extract. These evaluations were performed as follows:

***Alkaloids.*** 1 mg of extract was inserted into a test tube and then diluted in 1 mL methanol. Then a few drops of H _2_SO _4_ 1M was added. Afterwards, a few drops of Dragendorff reagent was added into the mixture. The formation of orange on filter paper indicated the presence of alkaloids.

***Flavonoids.*** 1 mg of extract was inserted into a test tube and diluted in 1 mL methanol. A few 2 mg of Magnesium powder was added followed by a few drops of concentrated HCl. The presence of flavonoids was identified by the formation of pink or red color.

***Phenolics.*** 1 mg of extract was introduced into a test tube and dissolved in methanol. Then a few drops of 1% FeCl _3_ were inserted. The formation of green, red, purple, dark blue or black indicated the presence of phenolics.

***Steroids and triterpenoids.*** 1 mL of methanol and 1 mg of extract were inserted into a test tube, stirred until homogeneous, then 2 drops of anhydride acetate and 1 drop of H _2_SO _4_ were added (Liebermann Burchard reagent). The formation of green or purple precipitation showed a sample containing steroids, and red precipitation displayed the presence of terpenoids.

***Saponins.*** 1 mg extract was put into a test tube and then dissolved in distilled water, and shaken strongly. The presence of saponins is characterized by the formation of durable foam on the surface of the liquid. Foam that remains stable after the addition of a few drops of concentrated HCl indicated the presence of saponins.

### Toxicity test

The toxicity test of extract was performed using brine shrimp lethality test (BSLT), as described previously ^12^. Methanol extract of bark of Tampoi ( *B. macrocarpa*) (1 mg) was dissolved using 100 μL of 1% DMSO (dimethyl sulfoxide) and homogenized. The samples were diluted using 150 μL of distilled water until the total of volume reached 250 μL, and then pipetted 200 μL and diluted again using 600 μL of distilled water until the total of volume was 800 μL, so that the sample concentration was 1000 ppm. Samples with a concentration of 500, 250, 125, 62.5, 31.2, 15.6, and 7.8 ppm were made from sample dilutions of a concentration of 1000 ppm. The control solution was made with the same treatment as the sample without the addition of extract.

The toxicity test was carried out using several standard micro plates. About 100 μL seawater containing 8-13 shrimp larvae was added to each diluted sample so that the sample volume was 200 μL (with a concentration of 500, 250, 125, 62.5, 31.2, 15.6, and 7.8 ppm). The number of dead shrimp larvae was calculated for 24 hours after treatment. Each sample was treated in triplicate. The data obtained was recorded and the value of LC _50_ calculated (Lethal Concentration 50%) using Probit analysis.

### Antioxidant assay

The antioxidant activity of the extract was evaluated using 2,2-diphenyl-1-picrylhydrazyl (DPPH) free radical scavenging method, as described previously ^11, 13– 15^. Briefly, the extract of bark of Tampoi ( *B. macrocarpa*) was prepared in a solution with a concentration of 25, 50, 75 and 100 ppm, respectively. 1 mL of extract and 1 mL of DPPH (0.024 mg/mL) were put into a test tube, which was incubated for 30 min at 37°C before being measured by Spectrophotometer UV Thermo Scientific Evolution 201 (measurements were carried out at a wavelength of 515 nm). Vitamin C was used as a positive control with variations in concentration: 2, 4, 6, and 8 ppm, respectively. Determination of antioxidant activity or DPPH scavenging effect (%) of extract and vitamin C were carried out in triplicates using equation as follow.

$$percentage\;\;of\;\;antioxidant\;\;activity=\frac{Absorbance\;\;of\;\;blank-Absorbance\;\;of\;\;sample}{Absorbance\;\;of\;\;blank}\times 100\%$$

Then, the value of IC _50_ (Inhibitory Concentration 50%) was determined using linear regression.

### GC-MS analysis

In order to obtain the information of the kinds of compounds in methanol extract of bark of Tampoi, an analysis using GC-MS 5977 was performed. Specification of column that used in this research was HP-5MS with length 30 m, diameter 0.25 mm, thick of film 0.25 μm. The identification of the compound was compared to NIST standard data ( https://webbook.nist.gov).

## Results

The secondary metabolites found in the methanol extract of the bark of Tampoi ( *B. macrocarpa*) are presented in Table 1.

**Table 1.** Phytochemical evaluation of the methanol extract of bark of Tampoi ( *Baccaurea macrocarpa*).

Secondary metabolites	Bark
Alkaloids	+
Steroids	+
Triterpenoids	+
Flavonoids	+
Phenolics	+
Saponins	˗

(+): Presence; (-): Absence

The result of toxicity test against *Artemia salina* larvae of the methanol extract of bark of Tampoi ( *B. macrocarpa*) can be seen in Table 2.

**Table 2.** Toxicity test of methanol extract of bark of Tampoi ( *B. macrocarpa*).

Average of three replicates performed for each concentration						
Concentration (ppm)	Log Concentration	Average of total larvae	Average of mortality	% Mortality	Probit	LC _50_ (ppm)
500	2.6989	10,3	2.3	22.3	4.23	1577.89
250	2.3979	10,7	2.7	25.2	4.33	
125	2.0969	10,3	3.3	32.0	4.53	
62.5	1.7959	10,3	1	9.7	3.66	
31.2	1.4948	10	4.3	43	4.82	
15.6	1.1938	9,7	0	0	0	
7.8	0.8928	9,3	2.7	29	4.45	

To evaluate the antioxidant activity of the methanol extract of the bark, DPPH method was performed. The results of the antioxidant test can be seen in Table 3.

**Table 3.** Antioxidant activity of the methanol extract of bark of *Tampoi* ( *Baccaurea macrocarpa*). Average of three replicates performed for each concentration.

Sample	Concentration (ppm)	Absorbance		Inhibition	Percentage of inhibition (%)	IC _50_ (ppm)
		Sample	Blank			
Bark	20	0.2190	0.4150	0.47229	47.229	11.15
	40	0.0560		0.88193	88.193	
	60	0.0490		0.86506	86.506	
	75	0.0305		0.92651	92.651	
Vitamin C	2	0.5470	0.6700	0.18360	18.360	3.28
	4	0.1530		0.77160	77.160	
	6	0.0450		0.93280	93.280	
	8	0.0340		0.94930	94.930	

**Figure 1.**
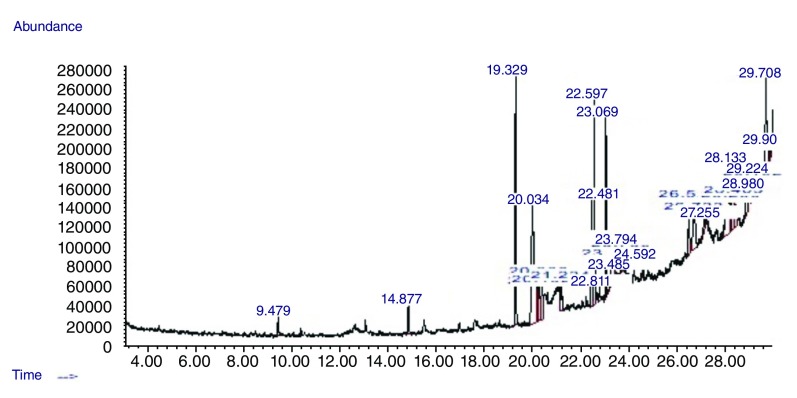
GC chromatogram of methanol extract of bark of *Tampoi* ( *Baccaurea macrocarpa*).

Furthermore, the methanol extract was analyzed using GC-MS analysis. The chromatogram and it compound contents of this extract is shown in Figure 1 and Table 4, respectively.

**Table 4.** Composition of compounds from methanol extract of bark of *Tampoi* ( *B. macrocarpa*).

Peak	Retention Time (min)	% Peak Area	Molecule Formula	Molecular Weight	Compounds
1	9.479	0.76	C _8_H _8_O _3_	152	Methylparaben
2	14.877	1.32	C _14_H _26_	194	Cyclohexane, 1-(cyclohexylmethyl)-2-methyl-, cis
3	19.329	9.91	C _17_H _34_O _2_	270.	Methyl palmitate
4	20.034	16.14	C _16_H _32_O _2_	256	palmitic acid
5	20.227	0.72	C _16_H _32_O _2_	256	palmitic acid
6	20.300	3.08	C _34_H _65_F _3_O _2_	562	Dotriacontyl trifluoroacetate
7	20.432	3.18	C _34_H _65_F _3_O _2_	562	Tricosyl trifluoroacetate
8	21.234	1.40	C _18_H _36_O _2_	284	Methyl 7-methylhexadecanoate
9	22.481	4.23	C _19_H _34_O _2_	294	9,12-Octadecadienoic acid (Z,Z)-, methyl ester
10	22.597	8.46	C _19_H _36_O _2_	296	9-Octadecenoic acid, methyl ester
11	22.811	0.62	C _29_H _60_O	424	Eicosyl nonyl ether
12	23.069	7.05	C _19_H _38_O _2_	298	Heptadecanoic acid, 16-methyl, methyl ester
13	23.334	3.34		336	Undec-10-ynoic acid, undecyl ester
14	23.431	0.29	C _18_H _32_O	264	9,17-Octadecadienal, (Z)-
15	23.485	0.07	C _24_H _48_O _2_Si	396	cis-Vaccenic acid
16	23.730	1.19	C _18_H _34_O _2_	282	Oleic Acid
17	23.774	1.15	C _15_H _24_O	220	(2S,3S,6S)-6-Isopropyl-3-methyl-2-(prop-1-en-2- yl)-3-vinylcyclohexan one
18	23.794	0.78	C _15_H _28_	208	7-Pentadecyne
19	24.592	0.67	C _18_H _35_ClO _2_	318	2- Chloropropionic acid, pentadecyl ester
20	26.520	2.77	C _21_H _42_O _2_	326	Methyl 18-methylnonadecanoate
22	26.733	3.58	C _20_H _42_	282	Eicosane
23	27.207	0.87	C _36_H _65_F _7_O _2_	662	Dotriacontyl heptafluorobutyrate
24	27.255	0.08	C _54_H _108_Br _2_	917	Tetrapentacontane, 1,54-dibromo-
25	28.234	0.74	C _28_H _58_	394	Octacosane
26	28.286	1.48	C _47_H _94_	659	Pentatriacontane, 13-docosenylidene-
27	28.374	2.31	C _19_H _36_	264	1H-Indene, 5-butyl-6-hexyloctahydro-
28	28.403	2.33	C _21_H _39_F _3_O _2_	380	Nonadecyl trifluoroacetate
29	28.941	1.68	C _29_H _52_	400	Nonacos-1-ene
30	28.963	0.31	C _22_H _41_F _3_O _2_	394	Eicosyl trifluoroacetate
31	28.980	0.34	C _23_H _46_	322	9-Tricosene, (Z)-
32	29.192	1.32	C _18_H _36_	252	1-Octadecene
33	29.224	1.10	C _26_H _52_	364	1-Hexacosene
34	29.708	7.09	C _23_H _46_O _2_	354	Methyl 20-methyl-heneicosanoate
35	29.829	0.10	C _18_H _36_	252	1-Octadecene
36	29.878	0.29	C _29_H _52_	400	Nonacos-1-ene
37	29.907	0.28	C _35_H _70_	490	17-Pentatriacontene

Sheet 1, raw data of the results of phytochemical evaluation for alkaloids, flavonoids, phenolics, steroids, triterpenoids, and saponins by observing the changing of colors; Sheet 2, raw data of the observation of the mortality numbers of *Artemia salina* Leach and calculation of LC _50_ value in toxicity test using brine shrimp lethality test; Sheet 3, raw data for antioxidant activity by DPPH method, including the measurement of absorbance using spectrophotometer in triplicates, the calculation of percentage of antioxidant activity, and the value of IC50; Sheet 4, raw data of GC-MS analysis Click here for additional data file. Copyright: © 2019 Erwin E et al. 2019 Data associated with the article are available under the terms of the Creative Commons Zero "No rights reserved" data waiver (CC0 1.0 Public domain dedication).

## Discussion

Based on the phytochemical evaluation, the results showed that the methanol extract of bark of Tampoi ( *B. macrocarpa*) contains several secondary metabolites including alkaloids, flavonoids, phenolics, steroids, and triterpenoids. Several secondary metabolites including alkaloids, steroids, triterpenoids, flavonoids, and phenolics are known to have antioxidant properties. These antioxidant compounds wield their activities through different mechanisms, for example by inhibiting hydrogen abstraction, radical scavenging, binding transition metal ions, disintegrating peroxides ^16, 17^, and one of the most important factors influencing antioxidant activity is the ability of the compounds to donate electrons.

Furthermore, in the present study the antioxidant activity of the Tampoi extract was determined by DPPH method. This method was used because it is simple, efficient, quick, more practical, and relatively inexpensive ^18^. Based on Table 3, it is known that the methanol extract of bark of Tampoi ( *B. macrocarpa*) can be categorized as an active extract in an antioxidant assay with IC _50_ value of 11.15 ppm. In addition, the results of the toxicity test using the BSLT method showed that the extract was not toxic because it displayed LC _50_ value above 1000 ppm ^12^.

According to the results of GC-MS analysis, the chromatogram showed 37 peaks (compounds). The profile of the compounds showed that the main components were fatty acids and fatty acid esters. Total content of unsaturated fatty acids and esters with a peak area of 19.88% including 9,12-octadecadienoic acid (Z,Z)-, methyl ester (peak area 4.23), 9-octadecenoic acid, methyl ester (peak area 8.46), undec-10-ynoic acid, undecyl ester (peak area 3.58), undec-10-ynoic acid, undecyl ester (peak area 3.346), cis-vaccenic acid (peak area 0.07), and oleic acid (peak area 0.19). It was been reported that unsaturated fatty acid compounds and unsaturated fatty acid esters have significant antioxidant properties ^19– 21^.

It can be seen that only a small part of those are aromatic compounds. However, aromatic compounds are compounds that have the ability to stabilize high free radicals. The mechanism of phenolics as antioxidants is started by the formation a bond between free radical (DPPH radical) and hydrogen atom from OH-phenolics (ArOH) to form ArO ^. ^radical. Hydrogen atom will easier to be released because of the presence of electron withdrawing group which is bound at *ortho*- or *para*- positions ^22^. Furthermore, ArO will react with a radical (ArO ^.^ or other radical) to form a stable compound ^23, 24^.

DPPH ^. ^+ AOH → DPPH-H + ArO ^.^

DPPH ^.^ + ArO ^.^ → DPPH-OAr or DPPH ^.^ + R ^.^ → DPPH-R

According to identification of the compound in the methanol extract of bark of *Tampoi* ( *B. macrocarpa*) using NIST database (DRUGBANK accession number, DB14212), it is known that the compound is identified as methylparaben. Based on the NIST database, peak at retention time at 9.479 min and peak area of 0.76% showed the characteristic of methylparaben (Molecular formula=C _8_H _8_O _3_; Molecular weight=152).

Methylparaben is widely used as a preservative in cosmetic products, medicines or pharmaceutical products and food ingredients ^25, 26^. and the antibacterial activity of methylparaben is stronger than benzoate acid ^27^. Methylparaben does not show negative effects on male mouse reproduction ^28^, but it was shown to have androgen antagonistic activity, to act as inhibitors of the sulfotransferase enzyme and to possess genotoxic activity. Paraben is allegedly able to trigger breast cancer in women ^29^.

**Figure 2.**
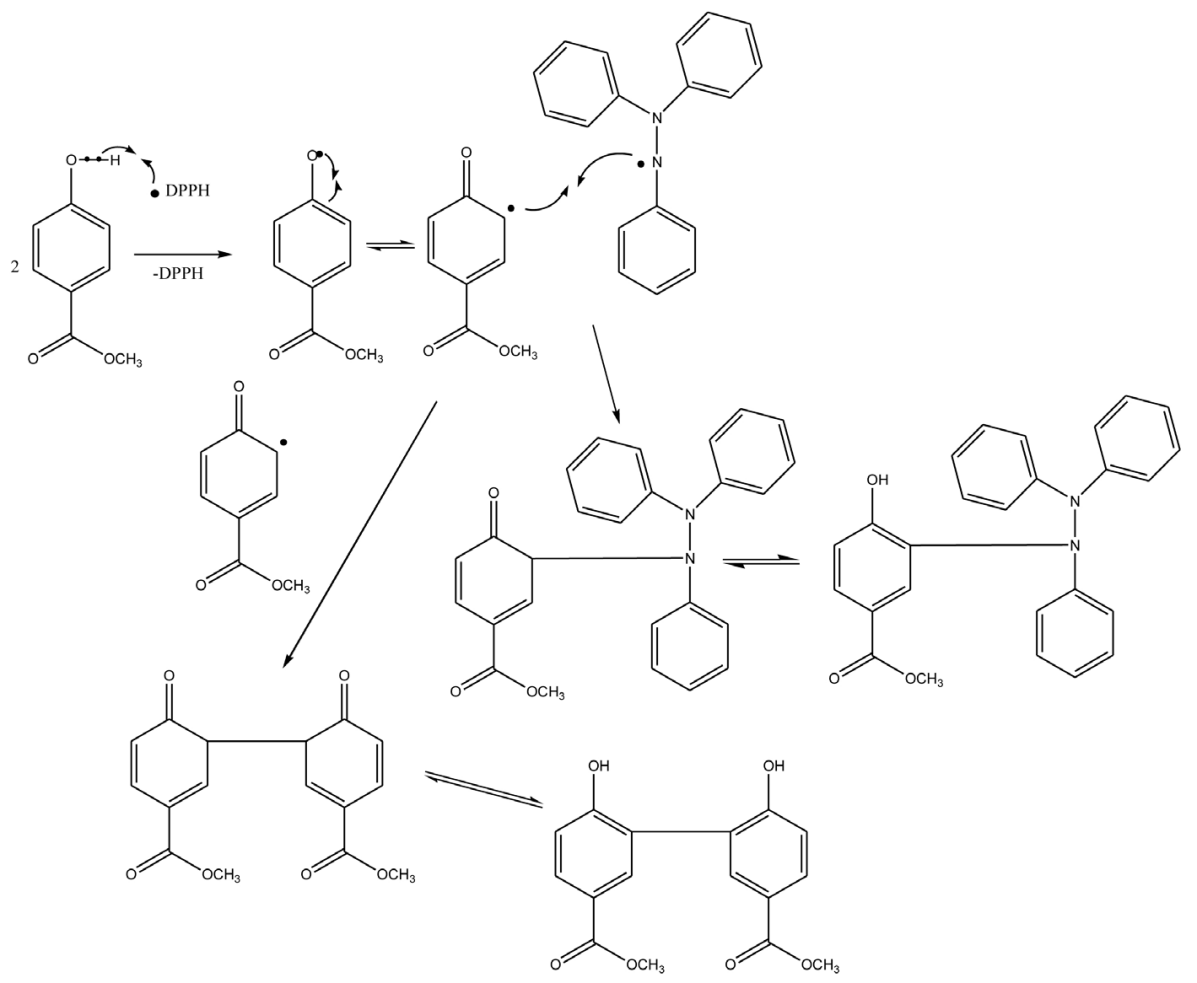
Prediction of DPPH radical scavenging mechanism by methylparaben.

Methylparaben is a phenolic group that can reduce free radicals because it contains aromatic groups, -OH clusters and carbonyl groups. The presence of –COOCH _3_ substituent at *para*- position in methylparaben makes this compound act as an electron withdrawing group. The bond dissociation energy (BDE) of the O–H bond is a main factor to investigate the action of antioxidant, due to the weaker OH bond the reaction of the free radical will be easier ^23^. As the prediction of the previous reaction mechanism ^11, 23^, the prediction of the reaction mechanism between DPPH radical and methyl paraben can be seen in Figure 2.

## Conclusion

The results of the study showed that the bark of Tampoi ( *Baccaurea macrocarpa*) has antioxidant activity with an IC _50_ value of 11.15 ppm.

## Data availability

The data referenced by this article are under copyright with the following copyright statement: Copyright: © 2019 Erwin E et al.

Data associated with the article are available under the terms of the Creative Commons Zero "No rights reserved" data waiver (CC0 1.0 Public domain dedication). 

F1000Research: Dataset 1. Sheet 1, raw data of the results of phytochemical evaluation for alkaloids, flavonoids, phenolics, steroids, triterpenoids, and saponins by observing the changing of colors; Sheet 2, raw data of the observation of the mortality numbers of Artemia salina Leach and calculation of LC _50_ value in toxicity test using brine shrimp lethality test; Sheet 3, raw data for antioxidant activity by DPPH method, including the measurement of absorbance using spectrophotometer in triplicate, the calculation of percentage of antioxidant activity, and the value of IC50; Sheet 4, raw data of GC-MS analysis., https://doi.org/10.5256/f1000research.16643.d227222 ^30^
