# Supplementary material for: GC-MS profiling and DPPH radical scavenging activity of the bark of Tampoi ( Baccaurea macrocarpa)
Source: F1000Res. 2019 Dec 12;7:1977. Originally published 2018 Dec 24. [Version 2] doi: 10.12688/f1000research.16643.2 (PMC6915813; doi:10.12688/f1000research.16643.2)
Supplement: Peer review history

# Peer review: Reviewer response for version 2

**DOI:** 10.5256/f1000research.23631.r57803

**Published:** 2019-12-20

**Authors:** Chanya Chaicharoenpong

**Affiliations:** 1 Institute of Biotechnology and Genetic Engineering, Molecular Crop Research Unit, Faculty of Science, Chulalongkorn University, Bangkok, Thailand

This manuscript reported the information of phytochemical and antioxidant activity of barks of *Baccaurea macrocarpa*.

1. The results of evaluation on phytochemicals of barks of *B. macrocarpa *showed that the methanol extract consisted of alkaloids, steroids, triterpenoids, flavonoids and phenolic compounds. But the profile of GC-MS showed only fatty acids, fatty acid esters and methyl paraben. The authors should use LC-MS to investigate chemical constituents of methanol extract instead of GC-MS.
2. Check the scientific name through the whole manuscript. When the scientific name appears first time in manuscript, it is full written both Genus and species, and then use the abbreviated binomial form for the following times.
3. In methods section, Phytochemical evaluation of alkaloids: “H _2_SO _4_ 1M” must correct to “1M H _2_SO _4_”
4. In methods section, Phytochemical evaluation of alkaloids: Please correct your statement “The formation of orange on filter paper….”
5. In methods section, Steroids and triterpenoids: “green or purple precipitation/red precipitation” must correct to “green or purple precipitate/red precipitate"
6. In methods section, the details of GC and MS conditions must clearly clarify.
7. In results section, the results should express your statistical analysis.
8. In results section, Table 2: It is essential to set the concentration of sample for calibration range to less than 50% mortality and greater than 50% mortality, and the calibration curve is linear. The results should express your statistical analysis.
9. In results section, Table 3: Check the data and calculation in Table 3. The results should express your statistical analysis.
10. In results section, Table 4: Data of peak no. 13 does not complete.
11. In discussion section, the data of GC-MS analysis (page 7) does not match the data in Table 4.
12. In discussion section, correct the equation of DPPH• and ArOH on page 7.
13. In discussion section, correct “benzoate acid” to “benzoic acid”.
14. To improve the quality of English, the authors must correct grammatical errors and English improvement throughout the manuscript.

I confirm that I have read this submission and believe that I have an appropriate level of expertise to confirm that it is of an acceptable scientific standard, however I have significant reservations, as outlined above.

# Peer review: Reviewer response for version 2

**DOI:** 10.5256/f1000research.23631.r57802

**Published:** 2019-12-16

**Authors:** Natthida Weerapreeyakul

**Affiliations:** 1 Division of Pharmaceutical Chemistry, Faculty of Pharmaceutical Sciences, Khon Kaen University, Khon Kaen, Thailand

I respectfully reject this manuscript as following reasons:

 This manuscript contains insufficient data although the authors tried to explain that this is a screening study.

 In addition to my previous comments I have following notifications:

- Table 2,  the LC _50 _value which is 1577.89 ppm was not in the concentration range (7.8 -  500 ppm) used in the study.
- Table 3, Inhibition should be rewritten.
- Table 3 , an IC _50_ value (11.15 ppm) of bark was suspicious as the lowest concentration used was 20 ppm.
- There is some typing error of DPPH reaction as AOH supposed to be ArOH.

I confirm that I have read this submission and believe that I have an appropriate level of expertise to state that I do not consider it to be of an acceptable scientific standard, for reasons outlined above.

# Peer review: Reviewer response for version 2

**DOI:** 10.5256/f1000research.23631.r57801

**Published:** 2019-12-16

**Authors:** Agustono Wibowo

**Affiliations:** 1 Faculty Applied Science, Universiti Teknologi MARA Pahang Branch, Jengka Campus, Pahang Darul Makmur, Malaysia

Approved.

I confirm that I have read this submission and believe that I have an appropriate level of expertise to confirm that it is of an acceptable scientific standard.

# Peer review: Reviewer response for version 1

**DOI:** 10.5256/f1000research.18189.r56216

**Published:** 2019-11-15

**Authors:** Chanya Chaicharoenpong

**Affiliations:** 1 Institute of Biotechnology and Genetic Engineering, Molecular Crop Research Unit, Faculty of Science, Chulalongkorn University, Bangkok, Thailand

This manuscript reported the new information of phytochemical and antioxidant activity of barks of *Baccaurea macrocarpa*.

 The authors used only DPPH assay to evaluate antioxidant activity. Antioxidant activity should be investigated using various assays to present antioxidant capacity of the extracts.

 The results of evaluation on phytochemicals of barks of *B. macrocarpa *showed that the methanol extract consisted of alkaloids, steroids, triterpenoids, flavonoids and phenolic compounds. But the profile of GC-MS showed only fatty acids, fatty acid esters and methyl paraben. The authors should use LC-MS to investigate chemical constituents of methanol extract instead of GC-MS.

 In results section, the authors did not mention on the toxicity test of the extract using brine shrimp lethality test. And the results should express yours statistical analysis.

 In discussion section, the third paragraph, the authors need to rewrite the total content and composition of fatty acids. GC chromatogram in Figure 1 was not related to the data of composition of compounds in Table 3 such as retention time, % peak area. For example, peak at retention time 19.329 showed high intensity on GC chromatogram but it expressed low % peak area just 0.91. The peak at retention time 14.877 showed low intensity on GC chromatogram but it expressed % peak area 1.32. Moreover, some compounds presented low matching percentage from the library searching. In Figure 2, structure of methyl paraben was wrong.

I confirm that I have read this submission and believe that I have an appropriate level of expertise to confirm that it is of an acceptable scientific standard, however I have significant reservations, as outlined above.

# Response

**Published:** 2019-11-21

**Authors:** erwin erwin

**Affiliations:** mulawarman university, Indonesia

Reviewer comment #1

The authors used only DPPH assay to evaluate antioxidant activity. The antioxidant activity should be investigated using various assays to present antioxidant capacity of the extracts. The results of evaluation on phytochemical of barks of *B. macrocarpa* showed that the methanol extract consisted of alkaloids, steroids, triterpenoids, flavonoids and phenolic compounds. But the profile of GC-MS showed only fatty acids, fatty acid esters and methyl paraben. The authors should use LC-MS to investigate chemical constituents of methanol extract instead of GC-MS.

Author response #1

This study is an initial screening of the antioxidant activity of Tampoi bark. The next project if we get the funding, we will use another antioxidant test and other instruments as you suggest. We will also carry out isolation and purification to obtain active compounds from Tampoi bark. Thank you for your valuable suggestion

Reviewer comment #2

In the results section, the authors did not mention on the toxicity test of the extract using brine shrimp lethality test. And the results should express your statistical analysis

Author response #2

Toxicity test data have been added in the revised article

Reviewer comment #3

some compounds presented low matching percentage from the library searching

Author response #3

It has been fixed

Reviewer comment #4

In Figure 2, the structure of methylparaben was wrong.

Author response #4

The structure of methylparaben has been fixed

Reviewer comment #5

In the discussion section, the third paragraph, the authors need to rewrite the total content and composition of fatty acids. GC chromatogram in Figure 1 was not related to the data of composition of compounds in Table 3 such as retention time, % peak area. For example, peak at retention time 19.329 showed high intensity on GC chromatogram but it expressed a low % peak area just 0.91. The peak at retention time 14.877 showed low intensity on GC chromatogram but it expressed % peak area 1.32.

Author response #5

The percentage (%) peak area of GC chromatogram has been fixed

# Peer review: Reviewer response for version 1

**DOI:** 10.5256/f1000research.18189.r56219

**Published:** 2019-11-11

**Authors:** Agustono Wibowo

**Affiliations:** 1 Faculty Applied Science, Universiti Teknologi MARA Pahang Branch, Jengka Campus, Pahang Darul Makmur, Malaysia

**Introduction:**

1. In first paragraph line 5, please correct your statement on natural ingredients “ do not contain chemicals” that only can be found in modern medicines, as all things in this world is formed from chemical constituents. Please change to “contain toxic chemicals”.
2. Last paragraph line 5, the statement “kinds of isolated compounds contained” are not correct as you don’t isolate the compound. Please remove the word “isolated”.

**Methods:**

1. DDPH assay alone can’t express the antioxidant properties of sample, so we suggest you to add other antioxidant assay such as ABTS and FRAP.

**Discussion:**

1. GCMS result indicated that the main constituent in *Baccaurea macrocarpa* extract is fatty acid, this is because the GCMS can only detect the volatile compounds. To identify other compounds that are responsible in the antioxidant activity of *Baccaurea macrocarpa*, we suggest you to run your sample using LCMS.
2. Methylparaben is familiar compound. Can you give literature which supported your claim that methylparaben is responsible to the antioxidant of *Baccaurea macrocarpa *extract?

I confirm that I have read this submission and believe that I have an appropriate level of expertise to confirm that it is of an acceptable scientific standard, however I have significant reservations, as outlined above.

# Response

**Published:** 2019-11-21

**Authors:** erwin erwin

**Affiliations:** mulawarman university, Indonesia

Reviewer comments #1

In first paragraph line 5, please correct your statement on natural ingredients “do not contain chemicals” that only can be found in modern medicines, as all things in this world is formed from chemical constituents. Please change to  “contain toxic chemicals”

Author response #1

Thank you for your suggestion, It has been fixed

Reviewer comments #2

The statement “kinds of isolated compounds contained” are not correct as you don’t isolate the compound. Please remove the word “isolated”

Author response #2

The word isolated has been removed

Reviewer comments #3

Methods:

DPPH assay alone can’t express the antioxidant properties of the sample, so we suggest you add other antioxidant assays as ABTS and FRAP

Author response #3

This study is an initial screening of the antioxidant activity of Tampoi bark.

Reviewer comments #4

**Discussion:**

GCMS result indicated that the main constituent in *Baccaurea macrocarpa* extract is a fatty acid, this is because the GCMS can only detect the volatile compounds. To identify other compounds that are responsible for the antioxidant activity of *Baccaurea macrocarpa*, we suggest you to run your sample using LCMS.

Author response #4

The next project if we get the funding, we will use another antioxidant test and other instruments as you suggest. We will also carry out isolation and purification to obtain active compounds from Tampoi bark.

Reviewer comments #5

Methylparaben is a familiar compound. Can you give literature which supported your claim that methylparaben is responsible for the antioxidant of *Baccaurea macrocarpa *extract?

Author response #5

I could not find in the literature that discusses the antioxidant properties of parabens in vegetation, but methylparaben is preservative and antioxidant in cosmetic products, medicines or pharmaceutical products, and food ingredients. Based on GC-MS data, methylparaben is most likely to be antioxidants

# Peer review: Reviewer response for version 1

**DOI:** 10.5256/f1000research.18189.r56217

**Published:** 2019-11-06

**Authors:** Natthida Weerapreeyakul

**Affiliations:** 1 Division of Pharmaceutical Chemistry, Faculty of Pharmaceutical Sciences, Khon Kaen University, Khon Kaen, Thailand

According to the GC chromatogram the peak that was claimed to be methyl paraben was detected at 9.479 min but when identified with the MS the peak at 9.467 min was identified instead. This might be wrong interpretation.

 Why the peak with high intensity detected at 19.329 min of methyl palmitate was not considered as the major compound or whether it was contributed to the antioxidant effect?

 Due to there are many antioxidant mechanisms, therefore, only DPPH scavenging activity is not sufficient.  

 Cytotoxicity result was not shown and sufficiently discussed in correlation with the antioxidant activity.

 The statistical analysis should be mentioned in the method section.

 Based on the insufficient information and evidence, this manuscript needs more experimentation and well written regarding the method, results and discussion.

I confirm that I have read this submission and believe that I have an appropriate level of expertise to state that I do not consider it to be of an acceptable scientific standard, for reasons outlined above.

# Peer review: Reviewer response for version 1

**DOI:** 10.5256/f1000research.18189.r44034

**Published:** 2019-11-04

**Authors:** Chinnadurai Immanuel Selvaraj

**Affiliations:** 1 Department of Biotechnology, School of Biosciences and Technology,, VIT University, Vellore, Tamil Nadu, India

The study design and the methodology sounds good. Still more details on the plant *B. macrocarpa *need to be included in the introduction. Even though the authors try to substantiate Methyl paraben as a non-toxic compound, it is a universally known fact usage of Methyl paraben is strongly discouraged in all human usage food and cosmetics as preservative. The Environmental Working Group (EWG) lists methylparaben as being a low to moderate Health Hazard. Parabens are potential endocrine disruptors due to their ability to mimic estrogen. Studies demonstrate that at sufficient concentrations, parabens can increase cell proliferation in human breast cancer MCF-7 cells, which are often used as a sensitive measure of estrogenic activity. Applying personal care product containing parabens—especially methylparaben—can lead to UV-induced damage of skin cells and disruption of cell proliferation (cell growth rate). These are evidenced reports on the Methyl paraben. Nevertheless, it is available in the natural source from the plant in meagre quantity. The authors can check for other compounds in GC-MS and state its importance in the manuscript. The GC-MS can be repeated. or HPLC can be performed using an aqueous extract. A simple TLC becomes handy for compound prediction, Then a column chromatography will be useful to check if there are any useful compounds.

 The authors fail to include the ill effects of Methyl paraben in the literature. Is there any reason for avoiding such inclusions? The authors should weigh the importance of other compounds in the plant. Is there any traditional/ancient usage of the fruit mentioned in literature must be included in Introduction section.

I confirm that I have read this submission and believe that I have an appropriate level of expertise to confirm that it is of an acceptable scientific standard, however I have significant reservations, as outlined above.

# Response

**Published:** 2019-11-21

**Authors:** erwin erwin

**Affiliations:** mulawarman university, Indonesia

Reviewer comment #1

The authors can check for other compounds in GC-MS and state its importance in the manuscript

Author response #1

We have re-checked the 37 peaks which appeared to be methylparaben most likely to be antioxidants 

Reviewer comment #2

The GC-MS can be repeated or HPLC can be performed using an aqueous extract. A simple TLC becomes handy for compound prediction, Then a column chromatography will be useful to check if there are any useful compounds.

Author response #2

This study is an initial screening of the antioxidant activity of Tampoi bark. The next project if we get the funding, we will use other instruments as you suggested. We will also carry out isolation and purification to obtain active compounds from Tampoi bark. We appreciated your suggestion

Reviewer comment #3

The authors fail to include the ill effects of Methyl paraben in the literature

Author response #3

We have added additional literature about the side effects of methyl paraben. Ref. no: 27

Reviewer comment #4

Is there any traditional/ancient usage of the fruit mentioned in literature must be included in the Introduction section

Author response #4

We did not find any use of tampoi as traditional medicine, but several other species of the genus *Baccuarea *were used as traditional medicine. In addition, there are several preliminary studies on Tampoi's bio-activity. Ref. no: 3,4,8, and 9
